# The International Congress

**Published:** 1904-01-15

**Authors:** Jas. Truman


### THE INTERNATIONAL CONGRESS.

BY JAS. TRUMAN, D.D.S.

The close of 1903 naturally brings the dental mind to the consideration of what may possibly take place during the coming year of 1904. The year just closing has been fruitful in many directions, and it is believed it has seen dentistry steadily advancing towards the goal that has been so strenuously worked for during many years prior to the present.

The most notable effort of 1903 has been the settlement of the controversy pending for over a year between the International Dental Federation and its committee appointed by the National Dental Association to arrange for the Congress of 1904. This controversy, which at one time was acute, has been amicably settled, and the preliminary work of the Congress is being pushed forward with an energy that gives every evidence of success. There was a small local cloud, not as "big as a man's hand," that hovered over St. Louis, but it is believed that even that has measurably been dissipated. Outsiders have had some difficulty in understanding what it was all about, but at no time did it endanger the success of the Congress. That has for its promotion a world-wide interest and can have nothing to do with local differences, only so far as a united local support is much to be desired, as it adds materially to the comfort of visitors.

At the present writing there will be but nine months in which to complete the preparation of the Congress, a time certainly limited for all that will be required. An effort should be made to secure papers of an original character. The old and tried subjects that have appeared at Congress after Congress, and convention after convention, might possiblv be consigned to obscurity. If there is one thing trying to the patience of the average regular attendant at conventions, it is to have served up repeatedly the professional dishes that have done duty so frequently as to be no longer mentally appetizing. If the committee on papers will use a wise discretion, they will not only eliminate these, but will equally limit the number prepared on any one subject. It is very possible that the Congress will be inundated with papers on inlays and porcelain. There should be a certain amount of this, but unless the papers presented have at least the flavor of originality, they might profitably be returned to the writers. The subject of porcelain in its various phases, while not yet perfected, may profitably be left to the masters of the art, and papers from these, in all lands, must always be acceptable.

The great need of dentistry at the present time is original work, and while it is true that there is more of this than at any former period, it is not sufficiently extended, and the literature, as presented in dental periodicals, is not, as a whole, of a character to increase the feeling of gratification with the progress of the profession.

If the Congress can be made a starting point for dentistry in the twentieth century, it will have accomplished much. The writer is, therefore, anxious that it shall prove, through its results, an incentive to scientific work. The younger generation of dentists need this encouragement. The product of the dental colleges throughout the world is of a higher character than at any former period, and its capability for greater results is equally pronounced. The character of the men having this in charge gives the assurance that this will be carefully guarded, and it is hoped that the suggestion may not be needed. It must be remembered, however, that it will be a serious disappointment if the Congress does not give us something to build upon before the time arrives for the next great international convention.

The world expects much of dentistry at this period in its history. It is recognized, as never before, as a prominent and important branch of the healing art, and our various educational means to this end must be continually advanced to meet this growing demand, and this applies with equal force to local, national, and international organizations.

Criticism in advance of performance is always an ungracious act, and the writer has no cause to exercise this function upon the preliminary work already accomplished. It has been entirely satisfactory, and gives promise of important results. It is, however, well to have the opinion of all shades of dental thought upon what is or is not expected of a congress such as this. The dentists of America are responsible for its success, and in order to accomplish this they must make an exhibit of dentistry as it stands to-day in this country. If we have anything that our *confreres* in other countries do not possess it is our duty to present it. Let our work speak for itself. No claim is made that American dentistry, if there be such a thing, is any better than dentistry the world over. This claim has never been made by dentists upon this side of the ocean, although it has frequently been charged as a fact, and a very discreditable fact it would be if true. That which the dentists of this part of the world do insist upon is that they have earnestly labored to unite dentistry into a progressive profession. Whether we are equal in this respect to other nationalities, or in advance of them, is a matter of no moment. It is believed that the dental world is rapidly growing, and narrow, contracted ideas are giving way to broader conceptions of professional duty and fraternal regard.

One of the important duties of a congress such as this is to foster the true cosmopolitan spirit, to unify the dental mind, and in this way mold the calling into a thoroughly-composite body in which the several parts are indistinguishable. It will probably always be impossible to establish standards of training acceptable to all nationalities, but it may be possible to reach definite conclusions as to what constitutes a cultured dentist, and when that has been accomplished it will not be based on an education strictly medical or upon a training strictly dental. To eliminate these two extremes is a part of the mission of international dental congresses. It is to be expected, and certainly it is earnestly desired, that the entire civilized world will send its delegates to St. Louis in September, 1904, and the assurance can be extended that they will receive a cordial reception, and, further, that the home organizations will earnestly co-operate in enlarging the field of observation and practice in dentistry, in order that it may become more and more in all nationalities a body of cultured, scientific men earnestly laboring, without selfishness, to lessen some of the ills of suffering humanity.—*International Journal.*

